# Metastasis-associated fibroblasts: an emerging target for metastatic cancer

**DOI:** 10.1186/s40364-021-00305-9

**Published:** 2021-06-10

**Authors:** Zimu Wang, Jiaxin Liu, Hairong Huang, Mingxiang Ye, Xinying Li, Ranpu Wu, Hongbing Liu, Yong Song

**Affiliations:** 1 Department of Respiratory Medicine, Jinling Hospital, Nanjing University School of Medicine, #305, East Zhongshan Road, 210002 Nanjing, Jiangsu China; 2 Department of Cardiothoracic Surgery, Jinling Hospital, 210002 Nanjing, China; 3 Department of Respiratory Medicine, Nanjing Drum Tower Hospital, Nanjing University School of Medicine, 210008 Nanjing, Jiangsu China; 4 Department of Respiratory Medicine, Jinling Hospital, Southeast University of Medicine, 210009 Nanjing, Jiangsu China

**Keywords:** Metastasis-associated fibroblasts, Cancer-associated fibroblasts, Metastatic cancer, Cancer treatment

## Abstract

Metastasis suggests a poor prognosis for cancer patients, and treatment strategies for metastatic cancer are still very limited. Numerous studies have shown that cancer-associated fibroblasts (CAFs), a large component of the tumor microenvironment, contribute to tumor metastasis. Stromal fibroblasts at metastatic sites are different from CAFs within primary tumors and can be termed metastasis-associated fibroblasts (MAFs), and they also make great contributions to the establishment of metastatic lesions and the therapeutic resistance of metastatic tumors. MAFs are capable of remodeling the extracellular matrix of metastatic tumors, modulating immune cells in the tumor microenvironment, promoting angiogenesis and enhancing malignant tumor phenotypes. Thus, MAFs can help establish premetastatic niches and mediate resistance to therapeutic strategies, including immunotherapy and antiangiogenic therapy. The results of preclinical studies suggest that targeting MAFs can alleviate the progression of metastatic cancer and mitigate therapeutic resistance, indicating that MAFs are a promising target for metastatic cancer. Here, we comprehensively summarize the existing evidence on MAFs and discuss their origins, generation, functions and related therapeutic strategies in an effort to provide a better understanding of MAFs and offer treatment perspectives for metastatic cancer.

## Background

Metastasis is an important cause of shortened survival of cancer patients and metastatic tumors remain largely incurable [1]. Metastasis is a complex multistage process, in which cells and other factors of primary tumors prime premetastatic niches (PMNs) in target organs, escape from primary sites, travel in the circulation and finally seed successfully in secondary tissues [2]. In addition to tumor cells themselves, other components in the tumor microenvironment (TME), which include cancer-associated fibroblasts (CAFs), extracellular matrix (ECM), endothelial cells and infiltrating immune cells, also play a significant role in the initiation and development of metastasis [3, 4].

CAFs are defined as fibroblasts associated with cancer, and they represent a dominant component of the tumor stroma [5]. Growing evidence has shown that CAFs can facilitate the progression of metastatic tumors by depositing and remodeling the ECM [6–9], thereby promoting the malignant phenotype of tumor cells [10–13], increasing the resistance of metastasizing tumors to current therapy [12, 14] and modulating other cells in the TME [15–18]. Stromal fibroblasts at metastatic sites can be termed metastasis-associated fibroblasts (MAFs) [19–21], and although they share many functions with CAFs in primary tumors, their effects on tumor progression are not equivalent, which may be caused by the organ milieu where they develop [22]. Compared with CAFs within primary tumors, MAFs have a stronger ability to augment the proliferation and migration level of tumor cells [23, 24], induce angiogenesis [19, 21] and suppress immune cells [23]. In addition, due to the very large differences in the environment where they develop, the origins and generation methods of MAFs and corresponding CAFs in primary tumors may be quite different (Table 1).

**Table 1:** Differences between CAFs at primary tumor sites and MAFs

Characteristics	Differences between CAFs at primary tumor sites and MAFs
Origins	• The sources of MAFs and CAFs at primary tumor sites vary according to the organ they locate at instead of the tumor type. • MAFs may originate from stromal cells from the primary tumor.
Generation	• MAFs can be generated before the arrival of metastatic tumor cells. • MAFs can be activated not only by adjacent tumor cells and other cells in the metastatic tumor microenvironment, but also by tumor cells and stromal cells at primary sites via extracellular vesicles.
Functions	• MAFs have stronger abilities of promoting tumor cells’ proliferation, migration, invasion and resistance to cytotoxic therapy. • MAFs decrease CD4^+^ T cell proliferation and suppress T cell activation more strongly. • MAFs are more highly activated in terms of extracellular matrix remodeling and stiffening ability and proangiogenesis ability. • S100A4^+^ CAFs inhibit tumor growth and angiogenesis at metastatic sites and not at primary tumor sites. • Since locations are different, the roles in the process of metastasis are different; for example, MAFs constitute an important part of premetastatic niche and create a friendly environment for metastatic tumor cells.

While the influence of CAFs has been extensively investigated in primary tumors, few studies have explored the role of MAFs in metastatic tumors, which are abundant within the metastatic microenvironment [25–29]. In this review, we will comprehensively summarize the available studies on MAFs and discuss their source, how they are generated, the way they function and potential therapeutic strategies targeting them and their related pathways.

### Origins of MAFs

CAFs are spindle-shaped cells in the TME that are negative for epithelial, endothelial and immune cell markers and lack cancer cell-specific mutations to exclude cells transformed from cancer cells via epithelial to mesenchymal transition (EMT) [30]. Due to the lack of CAF-specific markers, markers are usually combined to identify activated CAFs. The most common markers are α-smooth muscle actin (α-SMA) [15, 26, 31], fibroblast activation protein (FAP) [25, 31], fibroblast-specific protein 1 (FSP-1) [32, 33], vimentin [23, 33], and platelet-derived growth factor receptor-α (PDGFRα) [26, 33], which are also widely used. In addition, since CAFs are highly heterogeneous, certain markers are adopted to define subpopulations [34–36]. Similar to CAFs at primary sites, MAFs have been reported to be heterogeneous and can be divided into myofibroblastic MAF, growth factor and inflammatory gene-expressing MAF and portal fibroblast/mesothelial MAF populations according to a modified CAF single-cell RNA sequencing (scRNA-seq) signature [37].

**Fig. 1:**
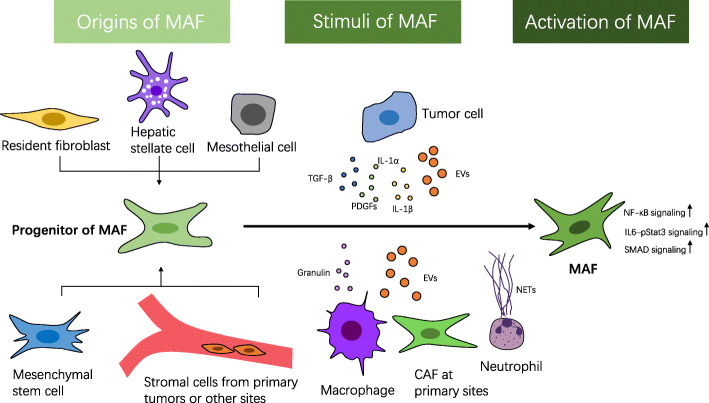
The potential origins and generation of MAFs. Possible progenitors of metastasis-associated fibroblasts (MAFs) are resident fibroblasts, hepatic stellate cells, mesothelial cells, mesenchymal stem cells and stromal cells derived from primary tumors or other sites. After receiving the stimuli from tumor cells or other cells, MAFs are activated. CAF, cancer-associated fibroblast; NETs, neutrophil extracellular traps; TGF-β, transforming growth factor-β; PDGFs, platelet-derived growth factors

Similar to CAFs in primary tumors, the origin of activated MAFs is not precisely defined. Possible cells of origin of MAFs include resident fibroblasts [25, 32, 38–40], hepatic stellate cells (HSCs) [26, 41, 42], mesenchymal stem cells (MSCs) [24, 43, 44], mesothelial cells (MCs) [45–47] and, of note, stromal cells derived from primary tumors [48–50] (Fig. 1). Generally, the source of MAFs is similar to the source of CAFs in the primary tumor at that site and varies considerably. Therefore, we will introduce the progenitor of MAFs according to the location of metastases.

#### MAFs in liver metastases

The liver is a common metastatic site for various tumors, such as colorectal cancer, pancreatic cancer, breast cancer and melanoma. Possible progenitors of MAFs in liver metastases are HSCs [26, 41, 42] and local fibroblasts [40], and they are not bone marrow derived [26, 40]. In an animal model of metastatic melanoma, predominant MAFs expressed glial fibrillary acidic protein (GFAP), which is an HSC marker and negative in local α-SMA-positive fibroblast-like cells, suggesting that MAFs may originate from HSCs [42]. Genetic tracing and scRNA-seq were performed by Bhattacharjee et al., who demonstrated that MAFs in liver metastases are primarily derived from HSCs; they found that over 90 % of MAFs are colocalized with HSCs and 80–91 % of MAFs strongly express an HSC signature [37]. The results of other studies support this speculation by showing that HSCs could differentiate into CAFs in vitro [26, 41]. Interestingly, the outcomes of another study showed that MAFs isolated from human colorectal liver metastases stain negative for markers related to HSCs, such as GFAP, desmin, or neural cell adhesion molecules, while they share the same markers with resident portal-located liver fibroblasts [40]. MAFs in colorectal liver metastases are also negative for CD45, a panleukocytic marker reported to be expressed by bone marrow-derived fibroblasts [51], indicating that they are not derived from bone marrow [40]. The outcome of a pancreatic ductal adenocarcinoma chimeric mouse model supports this speculation. Researchers engrafted tdTomato bone marrow into irradiated mice and found that MAFs in hepatic metastases were tdTomato-negative [26].

#### MAFs in lung metastases

MAFs are highly present in lung metastatic tumors [21, 25, 28, 38] and PMNs [52] and possibly originate from resident local fibroblasts [22, 25, 32, 38], bone marrow-derived MSCs [22] and CAFs from primary tumors [50]. Fibroblasts derived from resident lung fibroblasts and bone marrow-derived MSCs constitute MAFs in lung metastases of breast cancer, and the expression of PDGFRα can be used for differentiation [22]. Primary lung fibroblasts[25, 32] and established lung fibroblast cell lines [38] can be activated in response to certain stimuli, such as extracellular vesicles (EVs) from tumor cells [25, 38] or CAFs in primary tumors [32]. Incubation with conditioned medium from tumor cells can induce the differentiation of MSCs into fibroblasts, and an in vivo study indicated that the transition takes place within the metastatic microenvironment [22]. In addition, stromal cells derived from primary tumors may also be part of MAFs at metastatic sites [50]. In a mouse model, primary tumors with rich GFP^+^ stromal cell infiltration are generated and GFP^+^ cells positive for α-SMA and FSP-1 are detected in lung and brain metastases, indicating that these MAFs are from primary tumors [50]. Relatedly, CAFs detected in the circulation in the form of single circulating cells or CAF-circulating tumor cell clusters are correlated with cancer prognosis [53]. Similar results were also observed in a living zebrafish model [54].

#### MAFs in brain metastases

MAFs can be found in brain metastatic tumors, although fibroblasts are absent in normal brain tissue and primary brain tumors [24, 50]. Similar to MAFs in lung metastatic tumors, MAFs in brain metastases can be stromal cells from primary tumors, which has been discussed above [50]. CAFs isolated from primary breast cancer are able to promote brain metastasis in an environment of brain metastasis mimicked by two three-dimensional culture systems [49]. In another study, MAFs from human breast cancer brain metastases tested positive for STRO-1, a surface antigen expressed by bone marrow MSCs, and negative for GFAP, and they were able to differentiate into adipocytes, suggesting that they may originate from MSCs instead of cell types of the central nervous system [24].

#### MAFs in bone metastases

MAFs are also closely involved in the formation of metastatic bone lesions [27, 44, 48, 55]. Researchers have shown that MSCs from bone [43, 44] and primary tumor sites [48] may possibly be the progenitor of MAFs in metastatic bone tumors. Bone MSCs can be converted into MAFs in vitro [43, 44]. In an orthotopic murine xenograft model of breast cancer, MSCs migrated from the primary tumor to the bone marrow and then transitioned to MAFs [48].

#### MAFs in peritoneal metastases

While tumors frequently metastasize via blood or lymphatic vessels, abdominal tumors commonly disseminate through the peritoneal fluid and develop peritoneal metastases [46, 47]. Since the peritoneal cavity is lined by MCs, it is assumed that MCs are an important source of MAFs in peritoneal metastases, and this process is termed mesothelial-to-mesenchymal transition (MMT) [46]. On the one hand, α-SMA is coexpressed with mesothelial markers based on observations of human and mouse peritoneal biopsies with ovarian cancer metastases [46] and MCs isolated from the ascites of patients suffering ovarian cancer [45]. On the other hand, MCs show increased expression of CAF markers with an elongated morphology under the stimulation of tumor cells in vitro [46, 47]. In addition, fibroblasts dissociated from normal human omentum tissues can be activated into CAFs by ovarian cancer cells, suggesting that local fibroblasts are potentially another source of MAFs in peritoneal metastases [39].

### Generation of MAFs

The factors that contribute to the generation of MAFs are tumor cells and other cells from primary and secondary tumors (Fig. 1).

Communication with tumor cells can promote the generation of MAFs. In vitro studies show that the progenitors of MAFs become activated when incubated with conditioned medium from tumor cells [22, 42, 43, 52, 56]. A number of reports have demonstrated that ligands belonging to the transforming growth factor‑β (TGF-β) superfamily and platelet-derived growth factors (PDGFs) are capable of inducing the activation of CAFs[5], which has also been observed for MAFs [28, 39, 41, 45–47, 57, 58]. Tumor cell-derived TGF-β3 [28] and TGF-β1 [39, 41, 45–47, 57] can enhance the function of MAFs, possibly by upregulating SMAD signaling in MAFs [39, 45]. C-X-C chemokine receptor 4 (CXCR4) is important for the expression of MAFs in metastatic foci [59]. MAFs activated by TGF-β1 increase the secretion of stromal cell-derived factor-1 (SDF-1, also known as C-X-C motif ligand 12, CXCL12), which binds to CXCR4 of tumor cells and promotes their TGF-β1 production in turn [41]. Metastatic breast cancer cells educate fibroblasts in lung metastases by secreting interleukin-1 alpha (IL-1α) and interleukin-1 beta (IL-1β), which trigger nuclear factor‑κB (NF-κB) signaling in MAFs[20]. Similarly, prostate cancer cell-secreted IL-1β induces the transition of bone MSCs into CAFs in vitro and increases the expression of CAF markers in bone metastases in vivo [44]. Osteopontin derived from tumor cells is able to recruit MSCs from the primary tumor site and mediates their transition to MAFs within bone marrow [48]. The expression of cyclooxygenase-2 (COX-2) in breast cancer cells is positively correlated with the number and function of MAFs in metastatic lung nodules [60]. In hormonal therapy-resistant metastatic breast cancer, interleukin-6 (IL-6)/ phosphorylated signal transducer and activator of transcription 3 (pSTAT3) signaling is crucial for the proliferation and function of MAFs [61]. Under certain circumstances, direct cell-cell contact between tumor cells and MAFs is essential for the function of MAFs [39].

EVs are another important source of activating factors of MAFs [25, 38, 62, 63]. EVs are cell-derived membranous structures that include exosomes and microvesicles, and they enable intercellular communication by transferring lipids, proteins and genetic material [64]. MicroRNAs, such as miR-1247-3p, can be delivered by EVs from tumor cells to resident stromal cells, thereby activating MAFs by eliciting the NF‑κB signaling pathway [38]. Melanoma-derived EVs deliver mRNAs associated with the activation of inflammatory signaling to MAFs, thereby enhancing the proinflammatory and tumor-promoting functions of MAFs [63]. Proteins delivered by primary tumor cell-derived EVs, such as integrin beta-like 1 [25] and TGF-β1 [65], can help prime PMNs by activating MAFs in secondary organs via the NF‑κB pathway [25] or other pathways. Occasionally, tumor cell-derived EVs are incorporated into tumor-associated macrophages (TAMs) and transmitted to metastatic sites to create a prometastatic niche by inducing the conversion of MAFs, possibly via TGF-β1. Notably, when recipient cells are directly treated with tumor cell-derived EVs, conversion is not triggered, indicating that molecules from TAMs may have an important role in the uptake of EVs or activation of conversion-promoting factors [62].

Stimulation from other cells also contributes to the activation of MAFs; for example, MAFs can be activated by neutrophils via the formation of neutrophil extracellular traps, which is triggered by pancreatic cancer cells, thus promoting the formation of liver micrometastasis [66]. The loss of TGF-β signaling in osteoblasts is associated with an increase in MAFs in bone metastases, which possibly depends on the secretion of basic fibroblast growth factor [55]. TAMs, whose precursors are monocytes recruited from bone marrow by metastatic tumor cells, can produce granulin to increase the conversion of HSCs to MAFs in pancreatic ductal adenocarcinoma liver metastases [26]. Granulin is also crucial for MAFs’ function [26]. EVs derived from CAFs within primary tumors induce the activation of resident lung fibroblasts by enhancing TGF-β signaling via transferring thrombospondin-1 using EVs, thus creating a PMN [32]. In addition, other changes in the TME, such as intratumoral acidification [67] and hypoxia [42], can enhance MAFs’ functions.

### Functions of MAFs

**Fig. 2:**
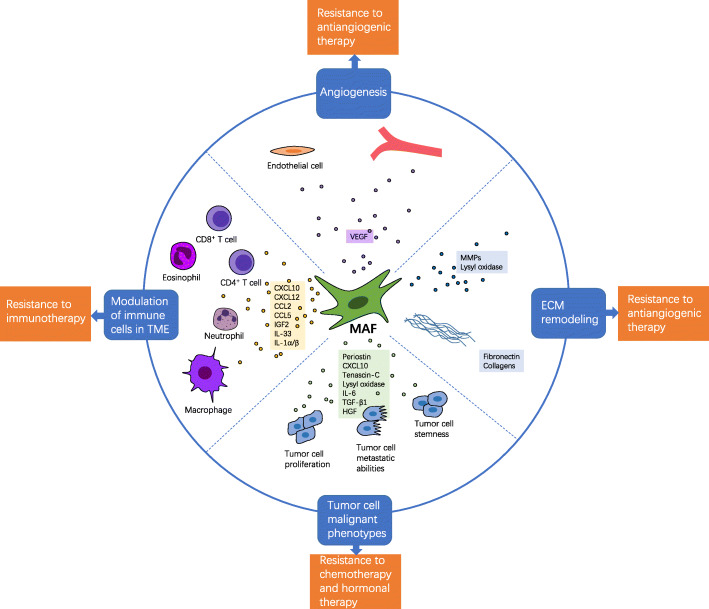
The functions of MAFs. After activated, metastasis-associated fibroblasts (MAFs) are able to remodel extracellular matrix (ECM), modulate immune cells in the tumor microenvironment (TME), induce angiogenesis and promote malignant phenotypes of tumor cells by expressing factors. With these abilities, MAFs are capable of mediating resistance to therapeutic strategies, including immunotherapy, antiangiogenic therapy, hormone therapy and chemotherapy. TGF-β, transforming growth factor-β; CXCL10, C-X-C motif ligand 10; CXCL12, C-X-C motif ligand 12; IL-1α/β, Interleukin-1 alpha/beta; MMP, matrix metalloproteinase; CCL2, C‑C motif chemokine ligand 2; CCL5, C‑C motif chemokine ligand 5; IGF2, insulin-like growth factor 2; IL-33, interleukin-33; VEGF: vascular endothelial growth factor; IL-6, interleukin-6; HGF: hepatocyte growth factor

The important role of MAFs in metastatic tumors may be achieved by creating a tumor-friendly microenvironment for metastatic tumor cells, enhancing the malignant characteristics of metastatic tumor cells and mediating resistance to therapeutic treatment (Fig. 2).

#### MAFs and metastatic TME

##### MAFs and ECM

The ECM in mammals is comprised of approximately 300 proteins, including collagen, proteoglycans and glycoproteins, and it is closely correlated with cancer progression and the regulation of angiogenesis and immune cell migration [68]. As the main component of the tumor stroma, MAFs can remodel the ECM in metastatic lesions by expressing factors such as fibronectin, TGFβR2 [22], collagen 1 [60], matrix metalloproteinase-2 (MMP2) [56] and other molecules. A study by Bhattacharjee et al. showed that type I collagen produced by MAFs restricts metastatic tumor growth mechanically and that this effect overrides MAFs’ stiffness-mediated tumor-promoting functions [37]. 

##### MAFs and immune cells

The tumor immune microenvironment (TIME), which includes innate and adaptive immune cells, exerts a tremendous influence on tumor progression and response to therapy [69]. CAFs modulate tumor immunity both directly and indirectly and are generally considered to promote a suppressive TIME [5]. Previous studies suggested that MAFs also suppress the TIME in metastatic lesions. A study by Chen et al. showed that metastatic breast cancer is devoid of CD8^+^ cytotoxic T lymphocytes and that CD3^+^ T lymphocytes are mainly located at the margin of metastatic tumors, which is dependent on CXCR4 signaling in α-SMA^+^ MAFs [31]. Inhibition of CXCR4 signaling decreases desmoplasia and reprograms the suppressive TIME of metastatic breast cancer, thus delaying the growth of metastatic tumors and improving survival in an animal model [31]. A similar pattern can be seen in a mouse model of gastric cancer peritoneal metastasis, in which MAFs were correlated with lower infiltration of CD8^+^ cells and higher infiltration of M2 macrophages [70]. A decrease in Foxp3^+^ regulatory T cells can be detected after MAF depletion [37]. Compared with CAFs in primary tumors, MAFs produce a higher level of C‑C motif chemokine ligand 2 (CCL2), CXCL12 and interferon-related genes, and have stronger abilities to decrease CD4^+^ T cell proliferation and suppress T cell activation by secreting insulin-like growth factor 2 (IGF2) [23]. In breast cancer, interleukin-33 (IL-33) is upregulated in MAFs but not in other cells in lung metastases, which facilitates recruitment of T cells and eosinophils to lungs and promotes type-2 immunity; moreover, the increase in IL-33 is significantly higher in lung metastases than other metastatic sites [71]. In addition, more neutrophils are recruited to melanoma lung metastatic niches after proinflammatory signaling is triggered in MAFs with elevated expression of IL-1α, IL-1β, CXCL10, CXCL1, CCL2, CCL3 and CCL5 [63]. In addition, MAFs can protect metastatic tumor cells from T cell-executed killing, which has been observed in a living zebrafish model [72]. The expression of chitinase 3-like 1 (Chi3L1) is upregulated in MAFs [73], and its inhibition can decrease lung metastases in a breast cancer model [74], suggesting that MAF-derived Chi3L1 may play a role in the metastatic microenvironment. CAF-derived Chi3L1 in primary tumors results in angiogenesis, macrophage recruitment, M2 macrophage phenotype and T cell exclusion; however, the mechanism by which it facilitates metastasis formation in secondary tumors still needs to be demonstrated [73]. 

##### MAFs and angiogenesis

Angiogenesis is an important hallmark of cancer associated with the need of oxygen and nutrients for tumor cells and the evacuation of carbon dioxide and metabolic wastes [75]. CAFs can positively regulate the angiogenesis of tumors [76]. Although patterns of tumor vascularization differ between primary tumors and metastatic tumors [76], MAFs also promote angiogenesis, which is consistent with CAFs within primary tumors. S100A4^+^ MAFs promote angiogenic microenvironment establishment in support of metastatic colonization by providing vascular endothelial growth factor-A (VEGF-A), while the ablation of S100A4^+^ CAFs does not affect angiogenesis at the primary tumor site [21]. Similarly, compared with CAFs isolated from primary CRC, MAFs in liver metastases induce more angiogenesis by cytokines, such as VEGF, and concomitant ECM remodeling, which trigger the activation of yes-associated protein/transcriptional coactivator with PDZ-binding motif (YAP/TAZ) signaling in endothelial cells; however, this phenomenon is not the same in colorectal cancer lung metastases [19]. Activated MAFs in liver metastases augment the expression of VEGF, which is mediated by COX-2, thereby inducing the migration and proliferation of hepatic sinusoidal endothelial cells and improving the level of angiogenesis within metastases [42]. MAFs in liver metastases of pancreatic cancer may promote angiogenesis and resistance to antiangiogenic drugs by providing CCL2 and CXCL8[77]. MAFs also play a role in the upregulation of angiogenesis in peritoneal metastases, possibly by secreting VEGF [46]. 

##### MAFs and PMNs

MAFs are an important part of PMNs, which are microenvironments established in remote organs by factors from primary tumors before tumor cells arrive at metastatic sites, facilitating the formation of metastatic lesions [78]. MAFs upregulate the expression of fibronectin in future metastatic sites to facilitate the adhesion of VLA-4^+^VEGFR1^+^ BMDCs, which play a crucial role in the modulation of PMN formation within tumor type-specific target organs [52]. MAFs can also promote the PMN formation by inducing ECM remodeling of metastatic organs by upregulating the levels of fibronectin, lysyl oxidase (LOX) and MMP9, and the increase in periostin might be a biomarker for this process [32]. In addition, MAFs secrete proinflammatory cytokines after activation, such as IL-6, IL-8 and IL-1β, and enhance the stemness and EMT phenotype of tumor cells to help them survive [25]. 

#### MAFs and metastatic tumor cells

MAFs can directly exert an effect on metastatic tumor cells and promote the development of their malignant phenotypes.

MAFs support the maintenance of cancer stem cells (CSCs), which play a key role in the process of metastatic colonization [79], by expressing periostin to recruit Wnt ligands and then elevating Wnt signaling in CSCs [28]. In addition, CAFs provide CXCL9 and CXCL10 to contribute to the CSC phenotype and proliferation of metastatic breast cancer cells, which bind to CXCR3 and activate JNK-IL-1 signaling in breast cancer cells; thus, a positive loop is established and MAFs are further activated [20]. S100A4^+^ MAFs in metastases attenuate apoptotic stress for tumor cells to support metastatic colonization by producing tenascin-C, an ECM protein that provides survival protection and likely functions via cooperative interaction with receptors or the promotion of the CSC phenotype [21].

MAFs are capable of promoting the proliferation [45] of metastatic tumor cells by secreting factors, such as periostin [26], PDGF [58], HA and hepatocyte growth factor (HGF) [37]. LOX is another factor produced by MAFs to enhance metastatic tumor cells’ proliferative level, which can reprogram glucose metabolism of metastatic tumor cells via the protein kinase B (AKT)-p70S6K/ hypoxia inducible factor 1 subunit alpha (HIF1α) pathway [57]. Compared with CAFs at the primary site, MAFs induce higher levels of proliferation, migration, invasion and drug resistance in tumor cells and promote their EMT and stemness phenotype, which is mediated by MAF-derived IGF2 [23]. IL-6 and IL-8 can also promote these phenotypes [38].

MAFs are able to enhance the adhesion, migration and invasion of metastatic tumor cells. For instance, an in vitro study showed that CAFs enhance the adhesion of breast cancer cells to brain microvascular endothelial cells and increase blood-brain barrier permeability, thus facilitating the transmigration of breast cancer cells and the establishment of brain metastases, which may be related to the upregulation of integrin α5β1 and αvβ3, αvβ3, c-MET and α2,6-siayltransferase in tumor cells [49]. The adhesion of tumor cells to the peritoneum is promoted by activated MAFs via enhanced β2-integrin-dependent tumor cell-MAF interactions rather than exposure of the underlying matrix [46], which may also be mediated by TGF-β1 [47], hepatocyte growth factor (HGF) and MMP2 [39]. Compared with CAFs from primary tumors, MAFs isolated from brain metastases secrete more CXCL16 and CXCL12, thereby attracting tumor cells to metastatic sites and promoting metastasis progression [24]. MAFs facilitate the metastatic tumor cell invasive phenotype in peritoneal metastases [46], possibly via TGF-β1 [47], HGF and MMP2 [39].

#### MAFs and drug resistance

MAFs can influence the efficacy of antiangiogenic therapy, and they contribute to angiogenesis and antiangiogenic therapy resistance in colorectal cancer liver metastases by increasing tissue stiffness [19]. MAFs mediate hormone therapy resistance in metastatic breast cancer by using EVs to transfer miR-221 to tumor cells and then convert these cells into CD133^hi^/ER^lo^/Notch3^hi^ CSCs, which are hormone therapy-resistant [61]. In addition, since MAFs contribute to a suppressive TIME via CXCR4 signaling, as discussed above, they promote the resistance of metastatic colorectal cancer to immune checkpoint blockade (ICB) therapy [31]. MAFs can also augment tumor cell resistance to chemotherapy drugs by producing cytokines, including IGF2 [23], IL-6 and IL-8 [38].

In addition, MAFs can also mediate some clinical symptoms of metastatic cancer. After activation by intratumoral acidification in bone metastases, MAFs express more inflammatory mediators (IL-6, IL-8 and CCL5) and nociceptive mediators (BNDF and NGF), which leads to hyperalgesia and ultimately continuous bone pain [67].

## Potential strategies targeting MAFs in metastatic cancer

At present, strategies that target MAFs to treat metastatic cancer can be divided according to two aspects: strategies that directly targeting MAFs and strategies that target mediators that play important roles in the upstream and downstream signaling of MAFs.

Strategies targeting MAFs themselves for the treatment of metastatic cancer are mostly limited to preclinical models. Direct depletion of MAFs suppresses desmoplastic metastatic tumor progression, and this effect of inhibiting metastatic tumor growth cannot be observed in nondesmoplastic metastases [37]. Although Bhattacharjee et al. found that MAFs have the effect of both inhibiting and promoting metastasis development, the depletion of MAFs significantly reduces metastases overall [37]. Another study depleted S100A4^+^ stromal cells in a mouse model, and pulmonary metastases were attenuated. Importantly, the authors identified that S100A4^+^ stromal cells, which are capable of facilitating metastasis, are most likely fibroblasts [21]. Regarding clinical trials, the most common target molecule of activated fibroblasts in the treatment of metastatic cancer is FAP. Since both MAFs and CAFs at primary sites express FAP, they may be both affected by these FAP-targeted strategies. Many clinical trials aim to explore the efficacy and safety of FAP-targeted therapy in various metastatic cancers (Table 2). Preliminary data show the potential antitumor activity of RO7122290, a FAP-targeted 4-1BB agonist, in combination with atezolizumab for patients with advanced solid tumors (objective response rate, 18.4 %) [80]. Three patients had objective responses for over 6 months when treated with RO6874281 [81]. However, another drug named sibrotuzumab failed to bring benefits to metastatic colorectal cancer patients, and progressive disease was observed in almost all patients [82]. In addition, ongoing clinical trials are evaluating the possibility of using FAP-related tracers to detect metastatic lesions (NCT04621435, NCT04457232, NCT04147494, NCT04459273, NCT04571086 and NCT04778345).

**Table 2:** Clinical trials targeting FAP in metastatic cancers

Study	Phase	Type of cancer	MAFs-related drug	Status	Outcome
NCT04826003	I/II	Metastatic Colorectal Cancer	RO7122290	Ongoing	Not applicable.
2017-003961-83	I	Advanced solid tumors	RO7122290	Ongoing	Objective response rate was 18.4 % when RO7122290 was used in combination with atezolizumab.
NCT00004042	I	Advanced or metastatic colorectal cancer	sibrotuzumab (F19)	Complete	Not reported.
Hofheinz 2003	II	Metastatic Colorectal Cancer	sibrotuzumab (F19)	Complete	Failed. Progressive disease was observed in all patients except for 2 patients with stable disease.
NCT03386721	II	Advanced/metastatic head and neck, oesophageal and cervical cancers	Simlukafusp Alfa (RO6874281)	Ongoing	Not applicable.
NCT02627274	I	metastatic head and neck cancer and breast cancer	Simlukafusp Alfa (RO6874281)	Ongoing	Preliminary data showed that objective responses over 6 months were observed in 3 patients.
NCT02558140	I	Locally advanced or metastatic solid tumors	RO6874813	Complete	Safety profile was favorable and preliminary antitumor activity was observed in 1 patient.

Since MAFs exhibit metastasis-promoting activity overall, approaches to suppressing their activation may be effective in treating metastatic cancers. CXCR3 is a key molecule in the interaction of breast cancer cells and MAFs, and the systemic administration of AMG-487, a CXCR3 antagonist, significantly suppresses pulmonary metastatic colonization in both immunodeficient and immunocompetent mice, which suggests that the antimetastatic activity of AMG-487 is mediated at least partially by the blockade of the activation of MAFs [20]. Another potential target is granulin, which is produced by TAMs at metastatic sites to increase the conversion to MAFs and activate MAFs, and an animal study showed that the depletion of granulin leads to decreased expression of MAFs and suppressed metastatic growth [26]. The IL-1β receptor antagonist anakinra is a potential useful drug for the treatment of prostate cancer bone metastasis, which can inhibit IL-1β -mediated recruitment and activation of MAFs, and its administration significantly impairs skeletal metastasis in an animal model [44]. Clinical trials exploring the role of anakinra in metastatic cancers are ongoing, including metastatic breast cancer (NCT01802970), metastatic colorectal cancer (NCT02090101) and various other metastatic cancers (NCT01624766). TGF-β1 signaling is another pathway of interest, and its inhibitor A83-01 was able to reduce fibrosis and impair peritoneal metastasis growth in a xenograft model [39].

Approaches focusing on downstream signaling pathways of MAFs also show antimetastatic functions. MAF-specific knockout of HGF and HAS2 decreases metastatic tumor growth and strongly extends the survival of mice, suggesting that HGF and HAS2 may be therapeutic targets for desmoplastic metastatic cancers [37]. Targeting IGF2, which can be secreted by MAFs to support metastatic tumor growth and modulate the TIME, along with its neutralizing antibody xentuzumab can inhibit the growth of MAF-tumor cell xenografts in vivo and may offer a novel therapeutic avenue for metastatic breast cancer [23]. Clinical trials are ongoing to study the effect of xentuzumab on metastatic cancers (NCT02123823, NCT03659136 and NCT03099174). Depletion of periostin, which MAFs produce in the metastatic niche to support stem cell phenotype and metastatic colonization, can decrease pulmonary metastases in an animal model [28]. BAPN, an inhibitor of LOX that is mainly secreted by MAFs in liver metastases, can markedly reverse LOX-mediated metastasis-promoting effects without significant toxicity [57]. Preclinical data suggest that the inhibition of IL-33 is another promising approach for metastatic breast cancer [71]. IL-33 is mainly produced by MAFs in lung metastases, and inhibition with its antibody significantly decreases the number and size of metastatic lesions [71]. Adeno-associated virus-mediated gene therapy can help target metastatic lesions more precisely, and Kobayashi et al. adopted it in a mouse model to augment BMP signaling in colorectal cancer liver metastases, which ameliorates metastatic tumors’ malignant phenotype and significantly improves survival without therapy-related liver injury [83].

Targeting MAFs also helps reverse MAF-mediated drug resistance in metastatic cancers. Inhibition of CXCR4 with AMD3100 resensitizes metastatic breast cancer to ICB by decreasing desmoplasia and thus reprogramming the immunosuppressive TME of metastases [31]. The results of a clinical trial (NCT02179970) prove that the CXCR4 inhibitor is able to induce an integrated immune response in metastatic lesions [84]. In addition, CXCR4 blockade reduces liver metastases in a mouse model [59]. Notably, αSMA^+^ cell-specific CXCR4 deletion significantly attenuates pulmonary metastasis, indicating the important role of MAFs in the antimetastatic activity of CXCR4 inhibition [31]. These findings indicate that CXCR4 is a promising target for metastatic cancer treatment, and clinical trials on metastatic cancer targeting CXCR4 are ongoing (NCT04177810 and NCT02907099). Suppressing MAF activation via renin-angiotensin system (RAS) inhibitors can decrease tissue stiffness and significantly enhance the efficacy of antiangiogenic therapy [19]. Moreover, clinical data indicate that that liver metastatic patients receiving antiangiogenic therapy with concomitant anti-RAS drugs have a longer overall survival than those treated with antiangiogenic therapy alone [19]. Breaking autocrine IL-6/Stat3 signaling with the IL-6 receptor inhibitor tocilizumab can reduce the expression of MAFs and restore sensitivity to hormonal therapy [61]. Although an IL-6 monoclonal antibody shows minimal effect as monotherapy in hormone therapy-resistant metastatic prostate cancer [85], clinical trials of other drugs targeting IL-6 in metastatic breast cancer (NCT03135171) and metastatic pancreatic cancer (NCT04191421 and NCT04581343) are underway to determine the effect of IL-6 signaling inhibition on metastatic cancers.

Taken together, MAFs exhibit prometastatic activity and mediate the drug resistance of metastatic cancers. Although strategies targeting MAFs show great potential in preclinical studies, there are differences between animal models and actual human conditions; thus, clinical trials are ongoing to test the safety and efficacy of these approaches in humans. Additional factors may also be of interest. First, compared with CAFs at the primary sites, there are relatively few studies on MAFs, and more research is needed for a better understanding of MAFs, including their subtypes and corresponding biological functions, to improve the efficacy of treating metastatic cancers. Second, although depleting MAFs can repress metastatic colonization, some MAFs can restrict metastasis growth mechanically [37]. It is important to determine the impact of long-term inhibition of MAFs’ antimetastatic function in therapies targeting MAFs themselves. Under such circumstances, it may be better to target downstream metastasis-promoting molecules directly or certain metastasis-promoting subtypes of MAFs than to target the whole population of MAFs. Third, since mice with some types of metastatic tumors benefit from anti-MAF therapy while mice with other types do not [37], screening of tumor types suitable for anti-MAF therapy is necessary.

## Conclusions

Metastasis accounts for a majority of cancer-related deaths[1]. Our review comprehensively demonstrates the role of MAFs in metastatic tumors. Generally, existing evidence shows that MAFs facilitate metastatic tumor development by promoting the establishment of metastatic sites and mediating therapy resistance. Understanding the crucial role of MAFs in metastatic tumors is of great significance for increasing the efficacy of treatment for metastatic tumors. Studies have shown that targeting MAFs and MAF-related pathways has an ideal effect on the treatment of metastatic tumors in terms of alleviating tumor metastases and reversing resistance to various therapeutic strategies. With a greater understanding of the role of MAFs and the development of novel therapeutic strategies, such as oncolytic virotherapy [86] and nanoparticle-based treatment [87], MAFs can be better targeted to tackle metastatic tumors in the future.

## Data Availability

Not applicable.

## Abbreviations

CAFs: Cancer-associated fibroblasts
MAFs: Metastasis-associated fibroblasts
PMNs: Pre-metastatic niches
TME: Tumor microenvironment
ECM: Extracellular matrix
EMT: Epithelial to mesenchymal transition
α-SMA: α-smooth muscle actin
FAP: Fibroblast activation protein
FSP-1: Fibroblast-specific protein 1
PDGFRα: Platelet-derived growth factor receptor-α
scRNA-seq: Single-cell RNA sequencing
HSCs: Hepatic stellate cells
MSCs: Mesenchymal stem cells
MCs: mesothelial cells
GFAP: Glial fibrillary acidic protein
GFP: Green fluorescent protein
MMT: Mesothelial-to-mesenchymal transition
TGF-β: Transforming growth factor-β
PDGFs: Platelet-derived growth factors
CXCR4: C-X-C chemokine receptor 4
SDF-1: Stromal cell-derived factor-1
CXCL12: C-X-C motif ligand 12
IL-1α: Interleukin-1 alpha
IL-1β: Interleukin-1 beta
NF-κB: Nuclear factor‑κB
COX-2: Cyclooxygenase-2
IL-6: Interleukin-6
pSTAT3: Phosphorylated signal transducer and activator of transcription 3
EVs: Extracellular vesicles
TAMs: Tumor-associated macrophages
MMP2: :Matrix metalloproteinase-2
TIME: Tumor immune microenvironment
CCL2: C‑C motif chemokine ligand 2
IGF2: insulin-like growth factor 2
IL-33: Interleukin-33
Chi3L1: Chitinase 3-like 1
VEGF-A: Vascular endothelial growth factor-A
YAP/TAZ: Yes-associated protein/transcriptional coactivator with PDZ-binding motif
LOX: Lysyl oxidase
CSCs: Cancer stem cells
AKT: Protein kinase B
HIF1α: Hypoxia inducible factor 1 subunit alpha
HGF: hepatocyte growth factor
ICB: Immune checkpoint blockade
RAS: Renin-angiotensin system
